# Decision-theoretical formulation of the calibration problem

**DOI:** 10.1155/S1463924689000143

**Published:** 1989

**Authors:** Miroslav Kárný, Katalin M. Hangos

**Affiliations:** 1 Institute of Information Theory and Automation Czechoslovak Academy of Sciences Pod vodárenskou věží 4 Prague 8 18208 Czech Republic; 2 Computer and Automation Institute Hungarian Academy of Sciences Kende u. 13/17 Budapest 1111 Hungary

## Abstract

The choice of calibration policy is of basic importance in analytical chemistry. A prototype of the practical calibration problem is formulated as a mathematical task and a Bayesian solution of the resulting decision problem is presented. The optimum feedback calibration policy can then be found by dynamic programming. The underlying parameter estimation and filtering are solved by updating relevant conditional distributions. In this way: the necessary information is specified (for instance, the need for knowledge of the probability distribution of unknown samples is clearly recognized as the conceptually unavoidable informational source); the relationship of the information gained from a calibration experiment to the ultimate goal of calibration, i.e., to the estimation of unknown samples, is explained; an ideal solution is given which can serve for comparing various ways of calibration; and a consistent and conceptually simple guideline is given for using decision theory when solving problems of analytical chemistry containing uncertain data. The abstract formulation is systematically illustrated by an example taken from gas chromatography.


Journal of Automatic Chemistry Vol. 11, No. 2 (March–April 1989), pp. 70–75

### Decision-theoretical formulation of the calibration problem

**Miroslav Kárný†**

*Institute of Information Theory and Automation, Czechoslovak Academy of Sciences, Pod vodárenskou věží 4, 18208 Prague 8, Czechoslovakia*

**and Katalin M. Hangos**

*Computer and Automation Institute, Hungarian Academy of Sciences, Kende u. 13/17, 1111-Budapest, Hungary*

*The choice of calibration policy is of basic importance in analytical chemistry. A prototype of the practical calibration problem is formulated as a mathematical task and a Bayesian solution of the resulting decision problem is presented. The optimum* feedback *calibration policy can then be found by dynamic programming. The underlying parameter estimation and filtering are solved by updating relevant conditional distributions. In this way: the necessary information is specified (for instance, the need for knowledge of the probability distribution of unknown samples is clearly recognized as the conceptually unavoidable informational source); the relationship of the information gained from a calibration experiment to the ultimate goal of calibration, i.e., to the estimation of unknown samples, is explained; an ideal solution is given which can serve for comparing various ways of calibration; and a consistent and conceptually simple guideline is given for using decision theory when solving problems of analytical chemistry containing uncertain data. The abstract formulation is systematically illustrated by an example taken from gas chromatography.*

### Introduction

The correct choice of calibration method has extreme importance in analytical chemistry and many papers can be found on this topic (see, for example, Hilton *et al.* [1], Kateman [2], Hunter and Lamboy [3]). The usual formulation of the calibration problem considers calibration as a separate preliminary phase of the measurement process which results in an estimate of some parameters of the calibration graph. In the course of the real measurement this calibration result is used only for the determination of the desired quantities from the measurement results.

In automated analytical measurements of large sequences of samples, the above two phases cannot be separated artificially. In automated laboratories it is common practice to insert known samples into a sequence of unknown samples in order to control the validity of the previous calibration and/or to re-calibrate the measurement system. There is a need to determine the optimum way of performing such checking and re-calibration. The aim of this paper is to propose a consistent formulation of this problem which enables the optimum calibration policy to be established.

† Author to whom correspondence should be addressed.

When analysing in depth this seemingly simple (at least from the statistical point of view) problem, we found that the full range of statistical decision theory is needed when formulating it. Much has remained to be done in order to construct a feasible suboptimum calibration policy as the optimum one is the well known non-feasible dual control design.

The paper is organized as follows. Based on the practical calibration task the exact formulation of the problem is given as a special case of the statistical decision model. The solution takes the form of dynamic-programming optimization. The optimization requires to solve identification and filtering problems is discussed in the final section before the conclusions.

The concepts in the paper are illustrated by a simple calibration example arising in gas chromatography. The parts related to this example are printed in *italics*.

### The practical calibration problem

A measurement device evaluates a set of samples. The result of the $t$th measurement is denoted by $y(t)$ and $t$ is called (discrete) time. The goal of the measurements is an estimation of a related quantity, say $x(t)$. The mapping

$$C: x(t) -- > y(t) \tag{1}$$

characterizes the measurement device and it is stochastic in nature. The measurement process is to be designed in such a way that relation (1) is: as deterministic as possible; (almost) invertible, allowing the construction of the backward mapping

$$C^{-1}: y(t) -- > x(t) \tag{2}$$

simple enough to be practically feasible; and stable through the time course.

Fairly often the deterministic constituent of the mapping $C$ is an (almost) linear function characterized by an absolute term and by a slope. The calibration problem arises when the mentioned pair or, more generally, a finite-dimensional vector of parameters $w$ determining fully the mapping $C$ is unknown.

The calibration is usually performed in such a way that to the sequence of samples with unknown values of $x(t)$, a portion is added with known values of $x(t)$ (control samples). The results of the calibration measurements are then used to specify $C$ more completely (by estimating the vector of unknown parameters and by substituting its point estimate for the unknown parameter vector).

0142–0453/89 $3.00 © 1989 Taylor & Francis Ltd.

*In gas chromatography, the tth measurement result $y(t)$ can be the area or the height of the peak corresponding to the component to be determined. The estimated quantity $x(t)$ is the concentration of that component.*

*The mapping C is the relationship between the measured peak area or the peak height and the concentration of the component. It is usually assumed to be a linear function (the case of linear calibration), i.e., of the form $y(t) = ax(t) + b + e(t)$ where a and b are constants and $e(t)$ is a random measurement error with zero mean.*

*The unknown vector parameter w consists of a, b and of parameter(s) characterizing the error distribution (typically, dispersion and mutual correlation of measurement errors for different samples).*

The calibration problem consists essentially of: the choice of calibration policy, that is, the choice of a rule which selects values of control samples and time instances at which the control samples are to be added; and the specification of how the results of measurements should be used when determining the value of the unknown sample. The Bayes solution given below solves (conceptually) both of these tasks.

### The formal calibration problem

This section 'translates' the calibration problem outlined above into the framework of the statistical decision model. We refer to De Groot [4] for more detailed information about this topic. The vital part of the solution, i.e., Bayesian identification (presented in control engineering terms), can be found in Peterka [5].

Our explanation consists essentially of the specification of key concepts of the decision theory, such as sample space, state space and decision space, in terms of the optimum calibration policy design. We start with the description of variables met throughout calibration. They are imbedded in three (overlapping) 'spaces' according to the way in which they influence the calibration and measurement process.

*Sample space*

A sequence of outputs $y(1...M) = (y(1),y(2),...,y(M))$ measured within a given finite-time horizon $M$ and the control samples $x(t_i)$ at checking time instances $t_i \varepsilon \{1,2,...,M\}$ are the sources of measured information for the calibration task, they form so called *sample space*. We assume that both the measured outputs $y(t)$ and inputs $x(t_i)$ are real scalars.

*In our example, the sample space consists of all measured data, i.e. of the peak areas or the peak heights and of the concentrations of the control samples.*

*State space*

The measurements $y(1...M)$ are fully determined both by the values for the samples $x(1...M) = (x(1), x(2),...,x(M))$ and by the measuring device (characterized by the mapping $C$). Possible $x(1...M)$ and mappings $C$ form so called *state space* of the calibration problem. To avoid subtleties of the non-parametric estimation, we assume that the mapping $C$ is completely characterized by a finite-dimensional vector of unknown parameters $w$, i.e., the state space consist of possible values of $x(1...M)$ and $w$.

Note that the control samples $\{x(t_i)\}$ belong both to the sample and state spaces, and are the directly measurable state entries.

*The concentrations of unknown and of control samples are the x-component of the sample space. The random mapping C is 'parametrized' by assuming that the case of linear calibration is relevant for gas chromatography and the measurement noise $e(1...M)$ has normal, mutually uncorrelated entries with a common dispersion r. The parameter vector completing the state of the problem is then $w = (a, b, r)$.*

*Decision space*

The triplet of decisions $u(t) = (u_1(t), u_2(t), u_3(t))$ have to be accepted at each moment $t$. The entries are given the following meaning:

$u_1(t)$: a known sample will be/will not be added (3)

$u_2(t)$: a value of the known sample chosen (4)

$u_3(t)$: an estimate of the unknown quantity $x(t)$ (5)

The sequences $u(1..M)$ of admissible values of $u(t)$ fill so called *decision space* and they are specified entrywise in the calibration case: $u_i(t) \varepsilon U_i$, $i = 1, 2, 3$.

*Any numerical value can be assigned to $u_1$. For instance, $U_1 = \{1, 0\}$ = {add control sample, do not add control sample} can be used. In practice, the admissible values of a known sample are given beforehand because standard, high-precision etalons are used for this purpose. Thus, $U_2$ = {values of available standards}. The choice of possible estimates from $U_3$ = {possible values of unknown concentrations} is natural.*

Any reasonable calibration policy has to balance its contribution to the measurement precision and its cost (in terms of time, money, energy, etc.). For automated design of the calibration, the degree of (dis)balance must be quantified. The following two elements of the discussed decision model are concerned with this aspect.

*Loss function*

The *loss function* $J$ is a scalar function of the data $d(1...M) = (d(1), d(2)..., d(M))$, $d(t) = (y(t), u(t))$ and of the state $x(1...M)$ (the parameter $w$ is not argument of the loss function as it is of secondary interest in the case treated). The following additive form of the loss is appropriate for the majority of the practical cases:

$$J(1...M) = J(d(1...M)), x(1...M)) = \sum_{t=1}^{M} q(d(t)) = \sum_{t=1}^{M} q(t) \quad (6)$$

The partial loss related to the $t$th measurement $q(t) = q(d(t), x(t))$ should usually be a smooth function of both arguments and a function bounded from below (hence it can be scaled to be non-negative).

The partial loss can often be structured as

$$q(t) = c(u_2(t))u_1(t) + (1 - u_1(t))\bar{q}(t)$$
$$= cu_1(t) + (1 - u_1(t))\bar{q}\,(u_3(t), x(t)) \quad (7)$$

where: the first term, given by the function $c(.)$, which depends generally on the value of the control sample $u_2(t)$, counts for the overall 'price' of the control sample (both price for the preparation of a control sample and the loss in the measuring productivity); and the second term, given by the function $\bar{q}(.,.)$, should reflect the loss due to mis-determination of the unknown sample. It is reasonable to assume that this function has its absolute minimum for the correctly determined value of the inspected sample, i.e., for $u_3(t) = x(t)$.

*Under the above widely acceptable assumptions, the loss $\bar{q}(.,.)$ measuring estimation error of an unknown sample can be approximated (using Taylor's expansion and the proper scaling) by the quadratic function*

$$\bar{q}(u_3(t), x(t)) = c_q(u_3(t) - x(t))^2 \quad (8)$$

*with an appropriate weight $c_q$.*

*Taken into account that the function $c(u_2(t))$ can often be approximated by a constant, the partial loss can be written in the simple form*

$$q(t) = cu_1(t) + (1 - u_1(t))(u_3(t) - x(t))^2 \quad (9)$$

*where the constant c has now the meaning of a relative price of the control sample cost versus the price of a particular error in the determination of the estimated concentration.*

*Criterion*

The loss function assigns values to various calibration and estimation outcomes after the measurement and it is not suitable for a prior comparison of possible calibrations and so is not suitable for the design purposes. For this, at the design stage, we shall minimize the expected value of the above loss function taking as the optimized criterion

$$K(1\ldots M \mid 0) = E[J(1\ldots M) \mid 0] \quad (10)$$

where $E[a \mid b]$ denotes the expected value of $a$ under the condition of $b$ and with the abbreviation

$$E[.\mid d(1\ldots t), b] = E[.\mid t; b] \quad (11)$$

In this notation, the definition (10) denotes the expected value of the loss function with no available measured information, i.e., with prior information only.

The expectation has to convert the loss function into the performance index, which can be evaluated *a priori*; it has to 'remove' all random and *unknown* quantities which prevent *a priori* evaluation of the consequences of the decision proposed. In this respect the unknown and random constituents of the relation $u(t) -- > y(t)$ are operationally indistinguishable. It supports the basic Bayesian postulate which states that the random and unknown quantities should be described in the same probabilistic way. this means that the expectation is applied both to random quantities in the usual sense and also to unknown parameters.

*In the illustrative example, the expectation is taken with respect to measurement noise $e(1\ldots M)$, generally randomized decisions $u(1\ldots M)$, unknown values of samples $x(1\ldots M)$ and unknown parameter $w = (a, b, r)$.*

The conceptual plausibility of the Bayesian design

$$K(1\ldots M \mid 0) -- >) \min \quad (12)$$

has the significant advantage that no policy exists which would achieve a uniformly smaller loss (irrespective of state realization) that does the Bayesian calibration policy.

*Admissible decision rules*

The last but very important part of the formalization specifies the *admissible decision rules.* When making decisions under uncertainty, the information available for it is the dominant optimization restriction.

The particular $u_i(t)$ can be chosen by a (generally random) mapping to the set $U_i$ from its information set:

$$u_1(t) : d(1\ldots t - 1) -- > U_1 \quad (13)$$

$$u_2(t) : d(1\ldots t - 1), u_1(t) -- > U_2 \quad (14)$$

$$u_3(t) : d(1\ldots t - 1), u_1(t), y(t) -- > U_3 \quad (15)$$

The above restrictions formalize that different entries of $u(t)$ can use different information sets. This fact can be explained easily if one takes into account the interpretations of the entries (see the discussion of the decision space). Note that this structure is not usual for standard formulations of decision tasks.

*Having measured the peak area/peak height data up to the time $t - 1$ we have to decide first if a known or an unknown sample will be measured next $[u_1(t)]$. The measured data can be used for this decision.*

*If we have decided to measure an unknown sample, i.e., if we know not only data up to the time $t - 1$ but also the value of $u_1(t)$, we have to choose the value of known sample concentration $u_2(t)$.*

*Selecting an unknown sample $[u_1(t) = 0]$, its estimation, i.e., the choice of $u_3(t)$, is additionally based on the measured sample value $y(t)$.*

*Summary of the formal calibration problem*

Having specified all elements of the formal calibration problem, we are ready to summarize its formulation, as follows:

1. Specify the loss function $J(1...M)$ which reflects the goals.
2. Specify the ranges $U_i$ of the decision variables $u_i$
3. Select as the (sub)optimum one the calibration policy consisting of the sequence of mappings (13), (14), (15) from the admissible information sets to the chosen decision ranges which (approximately) minimizes the expected values of the loss function.

### Conceptual solution of the calibration problem

The most effective procedure for the design of the optimum calibration policy, with the information restrictions of the type (13), (14) and (15) on admissible decision rules, is dynamic programming. We shall write the form which is specific to our case.

It can be shown that the optimum calibration policy can be taken as a non-randomized one. For any fixed data from the admissible information set the optimally chosen decision is the minimizing argument in the following functional (Bellman) equation:

$$K(t...M \mid t-1) = \min_{u_1(t)\varepsilon U_1, u_2(t)\varepsilon U_2} E\left[\min_{u_3(t)\varepsilon U_3} E\{q(t) + K(t+1...M \mid t) \mid t\} \mid t-1; u_1(t), u_2(t)\right] \quad (16)$$

this equation has to be solved recursively *backward:*

$$t = M, M-1, ..., 1 \quad (17)$$

starting with

$$K(M+1...M \mid M) = 0 \quad (18)$$

The term $K(t + 1...M) \mid t)$ denotes the function of data $d(1...t)$ which can be interpreted as the optimum expected loss-to-go (after the time $t$):

$$K(t+1...M \mid t) = \min E\left[\sum_{\tau=t+1}^{M} q(\tau) \mid t\right] \quad (19)$$

where the minimum is taken over admissible decision rules.

*When illustrating the above optimization we assume that the necessary moments of treated random variables are available. Their construction is described in the next section.*

*Starting the optimization for $t = M$ we should first minimize the inner expectation:*

$$\min_{u_3(M)} E[cu_1(M) + (1-u_1(M))(u_3(M) - x(M))^2 \mid M] = cu_1(M) + (1-u_1(M))dis(x(M) \mid M) \quad (20)$$

*where $dis(a \mid b) = E[(a - E[a \mid b])^2 \mid b]$ denotes the dispersion of the variable a conditioned on b. The minimum is achieved for $u_3(M) = E[x(M) \mid M]$.*

*When choosing $u_1(M)$, $u_2(M)$ we have to minimize the outer expectation*

$$\min_{u1(M)\varepsilon U_1, u_2(M)\varepsilon U_2} E[cu_1(M) + (1-u_1(M))dis(x(M) \mid M) \mid M-1; u_1(M), u_2(M)] \quad (21)$$

*For $u_1(M) = 1$ (add a control sample) we would have $K(M...M \mid M-1) = c$, irrespective of the value of the control sample $u_2(M)$.*

*For $u_1(M) = 0$ (measure an unknown sample) we would have*

$$K(M...M \mid M-1) = E[dis(x(M) \mid M) \mid M; u_1(M), u_2(M)] = E[(x[M) - E[x(M) \mid M])^2 \mid M-1]$$

*Even in this case, the value of the control sample $u_2(M)$ does not influence the result; it can influence the quality of the estimation of the calibration graph and consequently $dis(x(t) \mid t)$ for $t > M$ only.*

*The solution of the first stage consists of a simple comparison of the two possible values of the loss-to-go. It gives no guideline on how to choose the value of the control sample; therefore, at least because of this, the horizon $M > 1$ has to be used. The computational price is, however, substantial as the conditional moments involved, such as $E[(x(M) - E[x(M) \mid M])^2 \mid M-1]$, are, as a rule, complex functions of data with respect to which the expectation and minimization should be performed for $t = M-1, M-2, \ldots$. A more complete discussion of various aspects of the design as well as further references can be found in Kárný* et al. *[6].*

### State estimation for calibration purposes

The dynamic programming consists of the sequence of taking the conditional expectation and its minimization. Let us study which information is needed for these evaluations. The state estimation task will be formulated and also solved in this way. It splits in the measurement-output prediction which relies on the parameter estimation (called also identification) and in the estimation of unknown samples (called filtering).

Equation (16) contains the two different conditional expectations. We shall express them in terms of respective conditional probability densities c.p.d.f.s). When dealing with c.p.d.f.s, we shall use the short-hand notation similar to that used for the conditional expectation:

$$p(a \mid t; b) = p(a \mid d(1...t), b) \quad (22)$$

and particularly

$$p(a \mid t) = p(a \mid d(1...t)) \quad (23)$$

For a generic discrete time $t$ the inner expectation in equation (16) can be written as

$$E[. \mid t] = \int . p(x(t) \mid t) dx(t) \quad (24)$$

and the outer expectation as

$$E[. \mid t-1; u_1(t), u_2(t)] = \int . p(y(t) \mid t-1; u_1(t), u_2(t)) dy(t) \quad (25)$$

It exemplifies that a model building and a state estimation have to supply the *predictive* c.p.d.f.:

$$p(y(t) \mid t-1; u_1(t), u_2(t)) \quad (26)$$

and the filtering c.p.d.f.:

$$p(x(t) \mid t) = p(x(t) \mid t-1; y(t), u_1(t), u_2(t), u_3(t)) \quad (27)$$

where the last equality follows from the definition

$$p(. \mid t) = p(. \mid d(1\ldots t)) = p(. \mid d(1\ldots t-1), d(t)) = p(. \mid d(1\ldots t-1), y(t), u(t)) \quad (28)$$

As we search for strategies with $u_3(t)$ being the deterministic mapping specified by expression (15), this entry can be omitted in the condition of the filtering c.p.d.f. This makes it clear that both c.p.d.f.s under consideration are just factors in the chain-rule decomposition of the joint c.p.d.f. of $x(t)$ and $y(t)$. For further manipulations it is advantageous to deal with this joint c.p.d.f., which can be viewed as the *outer* probabilistic description of the calibration graph. The term 'outer' stresses that the description is based on the prior information and the measured data only. This model can be expressed as follows:

$$p(x(t), y(t) \mid t-1; u_1(t), u_2(t))$$

$$= \int p(x(t), y(t), w \mid t-1; u_1(t), u_2(t))\mathrm{d}w$$

$$= \int p(y(t) \mid t-1; x(t), w, u_1(t), u_2(t))$$

$$p(x(t) \mid t-1; w, u_1(t), u_2(t))p(w \mid t-1; u_1(t), u_2(t))\mathrm{d}w$$

$$= \int p(y(t) \mid x(t), w)p(x(t) \mid w, u_1(t), u_2(t))p(w \mid t-1)\mathrm{d}w \quad (29)$$

The two leading expressions in the above equation are implied by elementary calculus with c.p.d.f.s. The simplifications in the last step are a consequence of the assumptions which are discussed separately for each of the three factors involved. These c.p.d.f.s have definite meanings reflecting the modelling of the measurement process, the choice of measured samples and the Bayesian experience accumulation.

*The probabilistic model of the measurement process:* $p(y(t) \mid x(t), w)$

The output $y(t)$ of the measurement is assumed to be fully determined by the value of the measured sample $x(t)$ and by the parameters $w$. No additional information for output prediction can be gained observing the data $d(1\ldots t-1)$ and $u_1(t), u_2(t)$. In probabilistic terms, the pair $(x(t), w)$ is a sufficient statistics for output prediction.

The c.p.d.f. $p(y(t) \mid x(t), w)$ is the measurement model describing in probabilistic terms the mapping $C$ [equation (1)].

*Assuming our linear normal calibration case, the discussed c.p.d.f. has the form*

$$p(y(t) \mid x(t), w) = N_{y(t)}(ax(t) + b, r) \quad (30)$$

*where p.d.f.* $N_y(m, r)$ *of a normally distributed random variable* $y$ *with a mean* $m$ *and dispersion* $r$ *has the known form*

$$N_y(m, r) = \frac{1}{\sqrt{2\pi r}} exp\left[-\frac{(y-m)^2}{2r}\right] \quad (31)$$

*The probabilistic description of measured samples:* $p(x(t) \mid w, u_1(t), u_2(t))$

For the simplifications related to this c.p.d.f. we have used the fact that the type of samples chosen (unknown or control) is determined by $u_1(t)$. The value of the control sample coincides with $u_2(t)$. The unknown samples are assumed to be mutually independent with a fixed known (up to the parameter $w$) common c.p.d.f. $p(x(t) \mid w)$.

It should be noted that the samples could be assumed to be independent of $w$, i.e., of the measuring device. Sometimes, however, it is advantageous to relate parameters of the p.d.f. describing the distribution of $x(t)$ to the precision of the measuring device. Moreover, by extending the parameter vector $w$ by parameters characterizing distribution of the unknown samples, the incomplete knowledge of their distribution can also be faced. For simplicity, this approach will not be followed here.

The above consideration and assumption imply that the relevant sufficient statistics required are just the triple $(u_1(t), u_2(t), w)$. The explicit form of the discussed c.p.d.f. is

$$p(x(t) \mid w, u_1(t), u_2(t)) = u_1(t)\delta(x(t) - u_2(t)) + (1 - u_1(t))\, p(x(t) \mid w) \quad (32)$$

where $\delta(.)$ denotes the Dirac function.

*The newly introduced c.p.d.f. describes simply the distribution of an unknown sample typical for the measured concentrations. For instance,* $p(x(t) \mid w) = N_{x(t)}(\bar{x}, rR)$ *can be often used. The mean* $\bar{x}$ *and the multiple of the measuring device precision* $R$ *are either known or they extend the vector of unknown parameters of the problem.*

*Bayesian identification:* $p(w \mid t-1)$

Both of the assumed inputs $u_1(t)$, $u_2(t)$ are restricted to be deterministic functions of the observed history $d(1\ldots t-1)$; they provide no additional information about the parameters of the calibration graph and can be omitted from the condition.

The discussed c.p.d.f. contains available information about unknown parameters; it includes the results of the Bayesian estimation of the calibration graph (in the extended case it also identifies the population of the analysed samples).

The Bayes rule applied to the admissible decisions gives the following recursion for the c.p.d.f. under consideration:

$$p(w \mid t) \propto p(y(t) \mid t-1; w, u_1(t), u_2(t))p(w \mid t-1) \quad (33)$$

where $\propto$ denotes the proportionality up to a $w$-independent factor and the recursion starts with a user-supplied prior c.p.d.f. $p(w) = p(w \mid 0)$.

The model required for this recursion [note that $x(t)$ is missing in the condition] is given by

$$p(y(t) \mid t-1; w, u_1(t), u_2(t))$$

$$= \int p(y(t) \mid t-1; x(t), w) p(x(t) \mid w, u_1(t), u_2(t)) \mathrm{d}x(t) \quad (34)$$

where the above-discussed models and simplifications have been used.

*Applying the identification equations to our particular calibration case, we assume the known parameters $(\bar{x}, R)$ of the c.p.d.f. $p(x(t) \mid w)$ describing the distribution of the measured samples. In the calibration phase $[u_1(t) = 1]$, the sample value is known and equal to $u_2(t)$ so that equation (34) reduces to*

$$p(y(t) \mid t-1; w, u_1(t), u_2(t)) = N_{y(t)}(ax(t) + b, r)$$
$$= N_{y(t)}(au_2(t) + b, r) \quad (35)$$

*In the estimation phase $[u_1(t) = 0]$, the assumed normality of $p(x(t) \mid w)$ and equation (34) lead to*

$$p(y(t) \mid t-1; w, u_1(t) = 0, u_2(t))$$
$$= N_{y(t)}(a\bar{x} + b, r\,(Ra^2 + 1)) \quad (36)$$

*i.e., the studied c.p.d.f. is again normal with the unknown value of the sample $x(t)$ replaced by the population mean $\bar{x}$. Compared with the calibration step the dispersion is increased, depending on the slope and the dispersion ratio $R$.*

*Having the c.p.d.f. $p(y(t) \mid t-1; w, u_1(t), u_2(t))$, we are able to apply the Bayes equation (33). It is computationally advantageous to start it with the self-reproducing prior p.d.f. $p(w \mid 0)$ which guarantees $p(w \mid t)$ to have a fixed functional form. In the assumed normal case, the self-reproducing c.p.d.f. of unknown parameters is*

$$p(w \mid t) \propto r^{-\frac{v(t)}{2}}(Ra^2 + 1)^{-\frac{v_c(t)}{2}}$$
$$\exp\left\{-\frac{[-1,a,b]V(t,a)[-1,a,b]'}{2r}\right\} \quad (37)$$

*where the prime denotes transposition, the scalar $v(t)$ counts the overall number of samples and $v_c(t)$ the number of unknown samples. The (3,3) positive definite (information) matrix $V(t, a)$ evolves as follows:*

$$V(t,a) = V(t-1,a) + d'(t,a)d(t,a) \quad (38)$$

*using the data vector*

$$d(t,a) = [y(t), u_2(t), 1] \text{ for } u_1 = 1 \quad (39)$$

$$d(t,a) = \frac{[y(t), \bar{x}, 1]}{\sqrt{Ra^2 + 1}} \quad \text{for } u_1 = 0 \quad (40)$$

*The above quantities are to be given the initial conditions which express the user's available prior knowledge. The discussion of this choice is outside the scope of this paper and the reader is referred to Kárný [7].*

*The evaluation of the integral (29) would provide the solution of the experience accumulation in the calibration task. It is, however, numerically difficult owing to dependence of the matrix $V$ on the unknown parameter $a$. This is a consequence of the fact that not only control but also unknown samples are used for gaining information about the measuring device. We shall not enter into the computational details. Instead, the independence of the information matrix on the slope $a$ is reached by the crude approximation*

$$d(t,a) \approx d(t,\hat{a}(t-1)) \quad (41)$$

*where $\hat{a}(t-1)$ is an estimate of the slope $a$ based on the data $d(1\ldots t-1)$. Assuming this simplified case, the required c.p.d.f. (29) becomes a two-dimensional Student distribution [8], characterized by $v(t)$ degrees of freedom, by the expected value*

$$f = E\{[y(t), x(t)] \mid t-1; u_1(t), u_2(t)\} = \frac{[V_{13}, V_{23}]}{V_{33}} \quad (42)$$

*$[V = V(t-1, \hat{a}(t-2))]$ and the covariance matrix*

$$\mathrm{cov}\{[y(t), x(t)] \mid t-1; u_1(t), u_2(t)\} = \frac{(V_{yx} - V_{33}f'f)^{-1}}{(v(t-1) - 3)V_{33}} \quad (43)$$

*where $V_{yx}$ is the left-upper corner (2,2) submatrix of $V$.*

*The evaluation of the above characteristics can be shown to be formally equivalent to well known recursive least squares. Note that the measured data enter into the second moment in a very non-linear fashion (cf., discussion of dynamic programming).*

### Conclusions

The performed formalization of the calibration problem could be useful in the following directions: it clarifies the necessary models, assumptions and informational sources for the systematic solution of the problem; and it gives a conceptual solution of the problem which combines all informational sources (field and expert knowledge together with data) in a consistent way.

Application of the described methodology is far from trivial. It gives, however, guidelines both to experts in chemistry (which models are necessary and in which form) and associated mathematicians (the set of equations to be numerically handled is uniquely given). In our experience these clarifications are of such importance that they justify adding another paper to the literature dealing with calibration problems.

### References

1. Hilton, J., Talling, I. B. and Clark, R. T., *Laboratory Practice*, (1982), 990.
2. Kateman, G., *Trends in Analytical Chemistry*, **2** (1983), ix.
3. Hunter, W. G. and Lamboy, W. F., *Technometrics*, **22** (1981), 323.
4. de Groot, M. H., *Optimal Statistical Decisions* (McGraw-Hill, New York, 1970).
5. Peterka, V., in *Trends and Progress in System Identification*, Ed. Eykhoff, P. (Pergamon Press, Oxford, 1981).
6. Kárný, M., Halousková, A., Bohm, J., Kulhavý, R. and Nedoma, P., *Kybernetika Suppl.*, **21** (1985), No. 3–6.
7. Kárný, M., *Kybernetika*, **20** (1984), 376.
8. Zellner, A., *An Introduction to Bayesian Inference in Econometrics* (Wiley, New York, 1976).

